# Removal of zinc(II) from livestock and poultry sewage by a zinc(II) resistant bacteria

**DOI:** 10.1038/s41598-020-78138-z

**Published:** 2020-12-03

**Authors:** Jiang Huang, Jihong Wang, Lan Jia

**Affiliations:** 1 Key Laboratory of Straw Biology and Utilization, Ministry of Education, Jilin Agricultural University, Changchun, Jilin China; 2 College of Resource and Environment, Jilin Agricultural University, Changchun, Jilin China

**Keywords:** Biological techniques, Microbiology

## Abstract

In order to remediate Zn-contaminated livestock and poultry sewage, a zinc-resistant bacterial strain was screened and isolated from the manure of livestock and poultry and identified by molecular biology. The optimal conditions for removing zinc(II) from strain XZN4 were determined by single-factor experiments as follows: within 3 times of repeated use, pH value was 5, initial concentration of zinc(II) was 100 mg/L, the amount of bacteria was 6 g/L, the temperature was 25–30 °C, and the removal equilibrium time was 60 min. Then, through adsorption isotherm model, scanning electron microscope image, energy dispersive spectrum analysis, infrared spectrum analysis and sterilization control experiment, it was found that the removal of zinc(II) by bacteria was single-molecule layer adsorption, which was carried out in coordination with degradation. The influence of different concentrations of copper(II), ammonia nitrogen, phosphorus, and chlortetracycline on the removal of zinc(II) from livestock and poultry sewage by XZN4 strain in the actual application was discussed. The bacteria can reduce the concentration of zinc(II) from the complex livestock and poultry waste water to below the discharge standard, and has a strong environmental tolerance, the highest removal rate reached 88.6% and the highest removal amount reached 10.30 mg/L. The screening and application of XZN4 strain can thus be of great significance for the microbial treatment of zinc(II) in complex livestock and poultry sewage. The results will provide guidance for the microbial remediation of heavy metal pollution.

## Introduction

With the continuous development of agriculture, heavy metal pollution has threatened human health and adversely affected the stability of ecosystem^1,2^. zinc(II) (Hereinafter referred to as Zn^2+^) is another common heavy metal ion. Non-biodegradable, accumulated in human body; High concentrations can lead to physical diseases, which can damage liver, kidney function and immunity^3,4^. Currently, with the high dose of feed additives used for livestock and poultry breeding, the heavy metal content, such as Zn^2+^, in the sewage system has increased. Generally, the wastewater is enriched with complex harmful ingredients such as organic matter, heavy metals, and antibiotics, many of which affect the degradation of Zn^2+^, often requiring other treatments to meet the regulatory standards. Zn^2+^ has a high toxicity, difficult degradation, migration, and biological uptake, and after it enters the environment, it will not only cause harm to the environment, but also threaten human health and survival; it is therefore urgent to study the pollution and related remediation mechanisms^5^.

Microbial remediation technology has been used to reduce the toxicity of heavy metals and improve the environment by utilizing microorganisms with heavy metal resistance or adsorption and transformation properties^6^. Biosorption is one of the most promising remediation methods and has attracted much attention due to its environmental friendliness, high efficiency, cost-effectiveness, and large availability^7^. It has been proven that some microorganisms, including bacteria, fungi, and actinomycetes, and the extracellular polymers produced, can exhibit certain heavy metal adsorption capacity^8,9^. At present, most of the studies on the adsorption of heavy metals by relevant microorganisms have focused on Cd^2+^, Cu^2+^ and other elements, while the mechanism of adsorption of Zn^2+^ by relevant microorganisms has been less studied. For example, *Bacillus* can tolerate 1000 mg/L of Cd^2+^ isolated from municipal wastewater and is also resistant to other heavy metals such as Cr^6+^ and Ni^2+^^10^. Drew et al.^11^ discussed the Cu^2+^ adsorption mechanism of ammoxidation bacteria and archaea. Meanwhile, previous studies have shown the difficulty to screen qualified strains, such as Dave et al.^12^, with screened and isolated *Eichhornia* spp. The removal rate of Cu ions reached 85%, but the ultimate tolerance of this strain to Cu ions was not high enough. Andreazza et al.^13^ screened *Pseudomonas* sp. from a Cu contaminated vineyard soil. Although the strain had strong tolerance, its adsorption capacity was poor.

Zn^2+^ usually hinder the function of environmental microorganisms by destroying cell membrane and inhibiting enzyme activity^14,15^. Antimicrobial technology is different from general water treatment methods in many aspects. This method is more targeted, sustainable and eco-friendly, so it has attracted a lot of research attention^16,17^. Given this background, the main goals of this study were: Screening strains with strong tolerance, adsorption ability, and environmental resistance and optimize its application condition, the Zn^2+^ adsorption mechanism of an isolated bacteria and its application in complex livestock and poultry sewage was evaluated, so as to provide a theoretical basis for adsorbents with high selectivity and adsorption capacity and lay a foundation for the assessment of microorganisms in zinc pollution control.

## Results and discussion

### Identification of Zn^2+^ resistant strains

**Figure 1:**
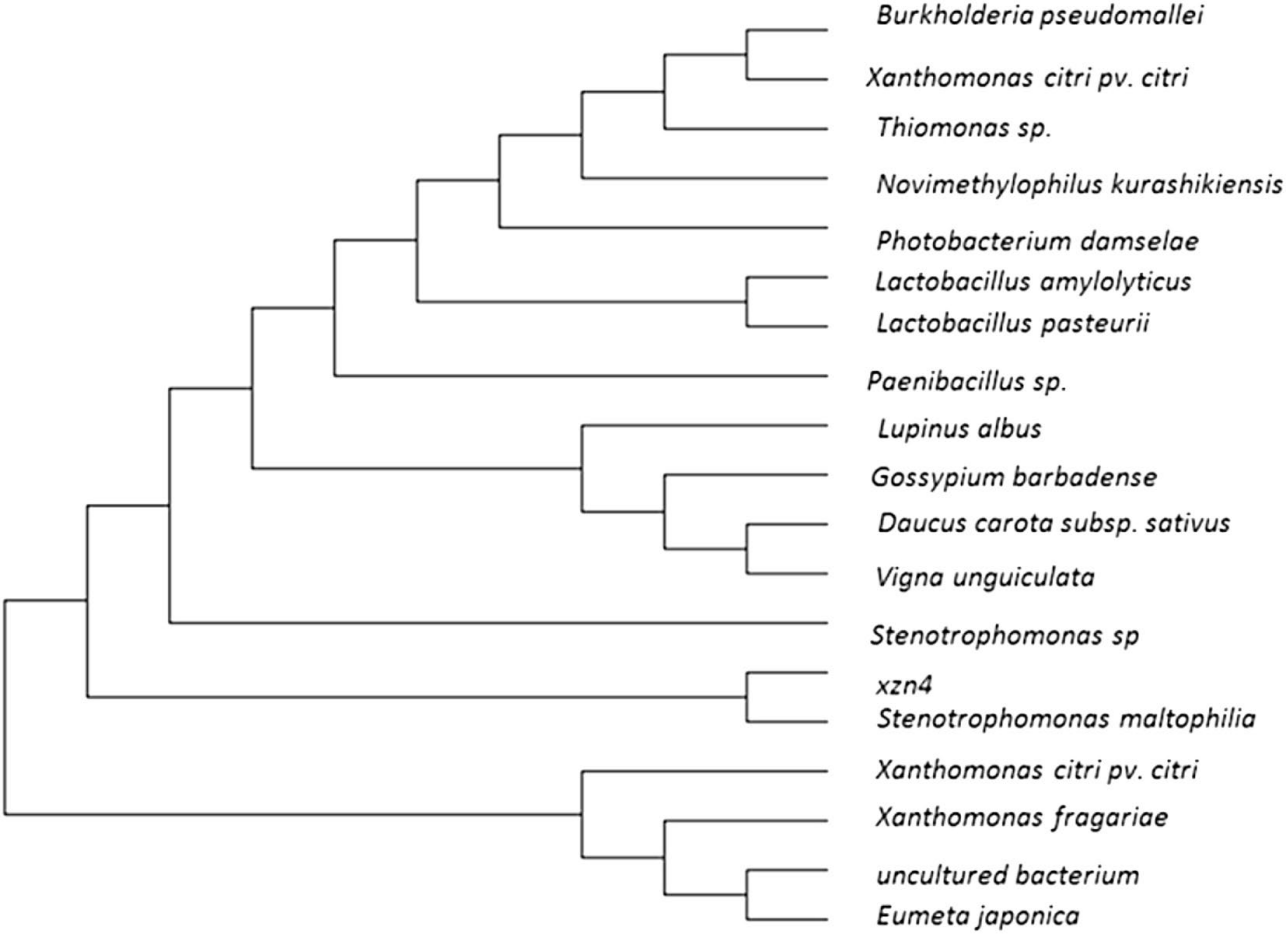
Phylogenetic tree of XZN4 strain.

The phylogenetic tree of the XZN4 strain is shown in Fig. 1. The results showed that the XZN4 strain gene sequence with eosinophilic malt narrow feed unit cell bacteria had 100% homology, thus belong to heavy malt narrow feed unit cell bacteria (*Stenotrophomonas maltophilia*).

### Study on the removal conditions of heavy metal Zn^2+^ by XZN4 strain

#### Tolerance of XZN4 strain to Zn^2+^

**Figure 2:**
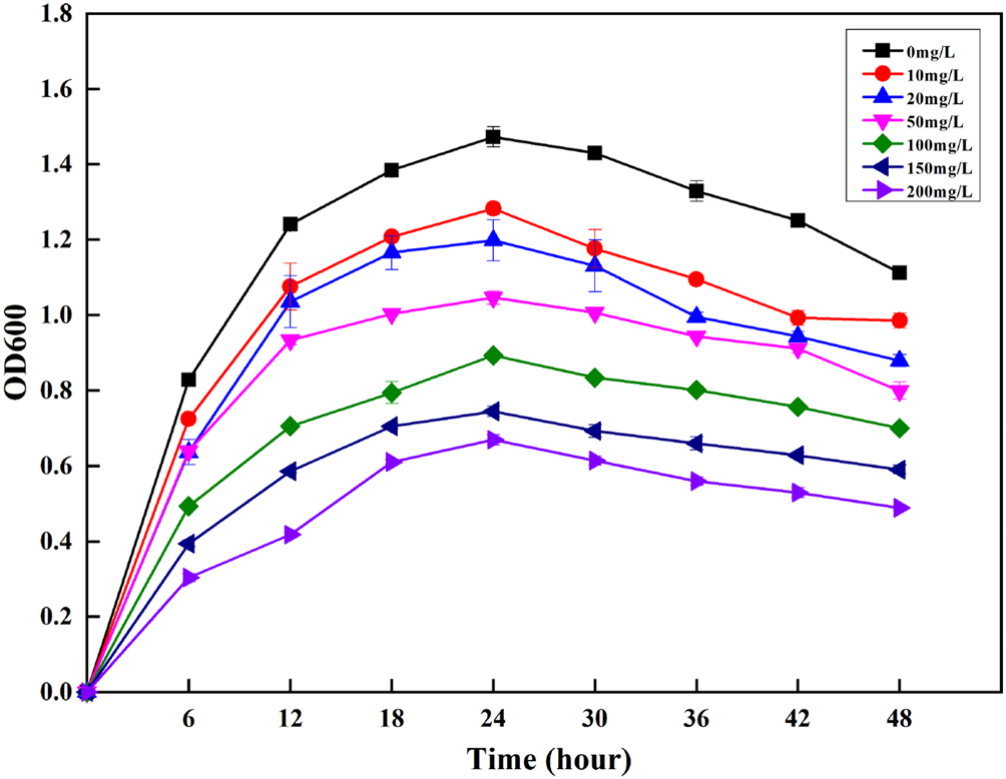
Growth of XZN4 strains at different concentrations of Zn^2+^. The initial concentration of Zn^2+^ was set to 0, 10, 20, 50, 100, 150, and 200 mg/L. Samples were taken every 6 h and the optical density (OD) value of the culture medium was determined at 600 nm.

The growth curve of the strain under different concentrations of Zn^2+^ is shown in Fig. 2, where the growth capacity of the bacteria gradually decreased with the increasing concentration of Zn^2+^. High concentration of Zn^2+^ could significantly inhibit the growth of the bacteria, but the growth did not completely stop, indicating that XZN4 bacteria had a strong tolerance to Zn^2+^.

When the concentration of Zn^2+^ in the sewage sample was 10–50 mg/L, the growth of the strain was not affected. When the concentration of Zn^2+^ in the sewage increased to 100–150 mg/L, the growth rate of the strain decreased and the optical density (OD) decreased gradually, indicating that the strain could tolerate a concentration of Zn^2+^ of 100 mg/L^18^. According to the classification by Mergeay et al.^19^, the XZN4 strain can be classified as moderately zinc-resistant bacteria.

#### Tolerance of XZN4 strain to pH

**Figure 3:**
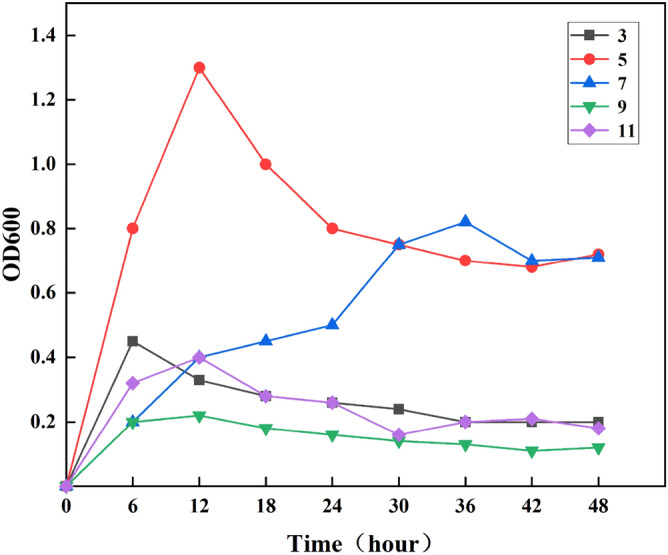
Growth of XZN4 strains at different pH. The initial pH was set to 3, 5, 7, 9, and 11. Samples were taken every 6 h and the optical density (OD) value of the culture medium was determined at 600 nm.

In this section, the growth of XZN4 strains with pH range of 3–11 were investigated. As can be seen from Fig. 3, strain XZN4 basically did not grow at pH was 3, 9, and 11, the growth of the strain was completely inhibited. Therefore, the optimal growth pH of XZN4 is within the range of 5–7. When PH was 7 showed that the growth retardation of the strain was prolonged, and it was possible that too high pH value would cause the change of cell charge. Therefore, through the above analysis, the optimal growth pH of strain XZN4 was 5.

#### Effect of repeated use on removal of Zn^2+^

With the increase of the use of microorganisms, the passivability will occur, and microbial biomass will also appear depletion. It can be found from Table 1 that the removal amount of Zn^2+^ by bacteria decreases with the increase of use times, and is positively correlated with the amount of microorganisms. When used for the fourth time, the ability of the bacteria to remove decreased by 60.4%, and the microbial population also decreased sharply, indicating that the maximum number of microbial replications was 3 times. When the number of repetitions reaches the fifth, the removal effect of microorganisms is very weak, and the microbial quantity is basically zero at this time.

**Table 1:** Effect of repeated use on the ability of the bacteria to remove Zn^2+^.

pH	1	2	3	4	5
Zn^2+^ removal rate (%)	81.1	73.5	65.8	32.1	14.2
Biomass (nmolP/g)	923	801	365	211	108

Biomass represents the number of microorganisms.

#### Influence of pH value on the removal of Zn^2+^

Levels of pH can affect the removal efficiency of microorganism, the chemical properties of the metal solution, and the activity of biological functional groups^20^. As shown in Table 2, when the pH value was 3, the removal rate of Zn^2+^ by XZN4 strain was low (16.9%). This is because under acidic conditions, the large amount of H^+^ would protonize the functional groups on the cell wall surface^21^. With the increased pH of the solution, the degree of protonation of functional groups on the cell wall surface will gradually decrease, the binding of active sites will increase, and the removal rate will continuously increase. Optimal removal rates were obtained at a pH between 5 and 6. When the pH value was 5, the removal rate reached the maximum removal rate of 81.5%. When the pH value exceeded 6, the removal rate gradually decreased, because OH^–^ will gradually form in the solution causing hydroxide precipitation with heavy metal ions in the solution, which will hinder the adsorption process. Therefore, the optimum pH value for removing Zn^2+^ was 5.

**Table 2:** Effect of pH on the ability of the bacteria to remove Zn^2+^.

pH	3	4	5	6	7	8
Zn^2+^ removal rate (%)	16.9	43.6	81.5	68.5	65.6	62.52

#### The influence of the bacterial concentration on the removal of Zn^2+^

**Figure 4:**
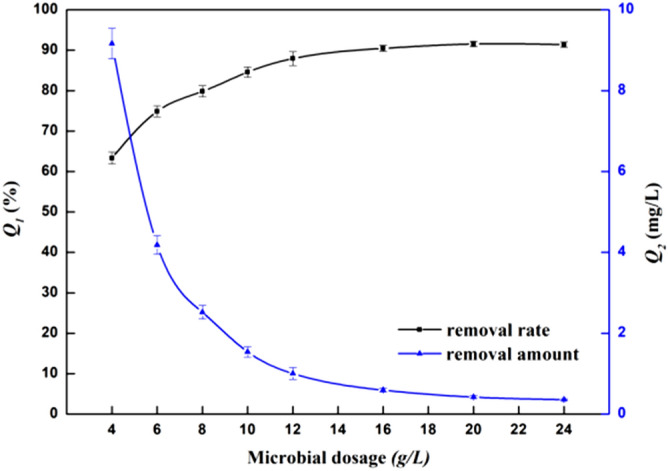
Effect of microbial dosage on the ability of bacteria to remove Zn^2+^. Q_1_ is the removal rate (%), and Q_2_ is the removal amount (mg/L).

Figure 4 shows the significant influence the amount of the bacterial strain had on the removal of Zn^2+^. The removal rate of Zn^2+^ increased with the increased amount of bacteria (4–20 g/L). At this concentration, the metal ions in the solution will not only be adsorbed by the surface of the microorganism, but also improve the removal rate by promoting the intracellular concentration of the metal ions^22^. The highest removal rate of Zn^2+^ was 91.6% when the amount of bacteria was 20 g/L. The removal rate of Zn^2+^ decreased gradually with the increased bacterial content. With a bacterial content between 4 and 12 g/L, the rate of decrease was faster. Meanwhile, when the amount of bacteria was between 12 and 24 g/L, the rate of Zn^2+^ decrease was slow. The amount of Zn^2+^ and the binding sites can be increased by appropriately increasing the amount of the bacteria, so as to improve the removal rate. However, with the increasing concentration of bacteria, the binding site between the bacteria and the heavy metal reached saturation, and the effective adsorption points were occupied. The removal rate gradually balanced, and the removal amount began to decrease. In order to maximize the microbial utilization rate, the equilibrium point of removal rate and removal amount was selected as the optimal amount of bacterial addition^23^. Therefore, the most appropriate addition amount of bacteria was 6 g/L, with a removal rate of 74.9% and a removal amount of 4.1833 mg/g.

#### Influence of initial concentration of heavy metal on the removal of Zn^2+^

**Figure 5:**
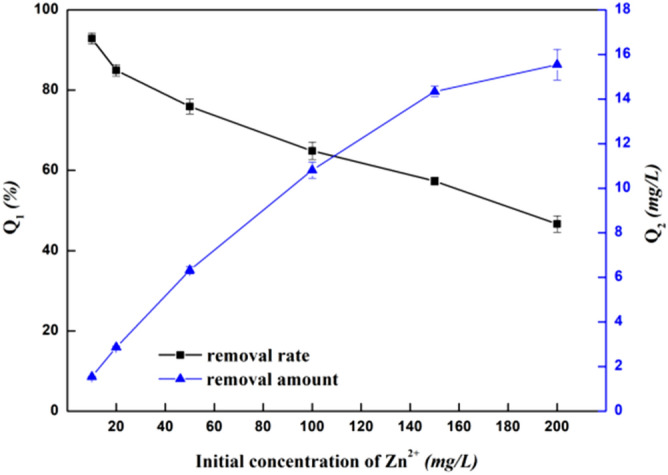
Effect of initial concentration of Zn^2+^ on the ability of bacteria to remove Zn^2+^. Q_1_ is the removal rate (%), and Q_2_ is the removal amount (mg/L).

The influence of the initial concentration of heavy metal ion on the removal of Zn^2+^ is shown in Fig. 5. When the initial concentration of Zn^2+^ was 10 mg/L, the removal rate was 92.8% and the removal amount was 1.5472 mg/g. At this time, because the initial concentration of heavy metals was low, the removal amount was low, but the ions can quickly bind to the bacterial surface and the removal rate was high. With the increase of Zn^2+^ concentration, the removal rate decreased gradually while the removal amount increased gradually. When the concentration of Zn^2+^ was between 25 mg/L and 100 mg/L, the amount of removal increased rapidly. When the concentration of Zn^2+^ was between 100 and 200 mg/L, the increase was relatively slow. Within a certain range, and with the increased concentration of heavy metal Zn^2+^ in the solution, the number of Zn^2+^ that the bacteria can bind to will also increase, so that the removal amount is significantly increased. However, when the concentration of heavy metal Zn^2+^ in the solution exceeds a certain range, the removal amount of Zn^2+^ tends to be balanced as the binding site between the bacteria and Zn^2+^ reaches saturation. In order to achieve the optimal Zn^2+^ removal rate and amount, the intersection point of the two was selected as the optimal removal condition for the initial concentration^24^. Therefore, the optimal initial concentration of Zn^2+^ was 100 mg/L with a removal rate of 64.9%, and a total Zn removal of 10.8111 mg/g.

**Figure 6:**
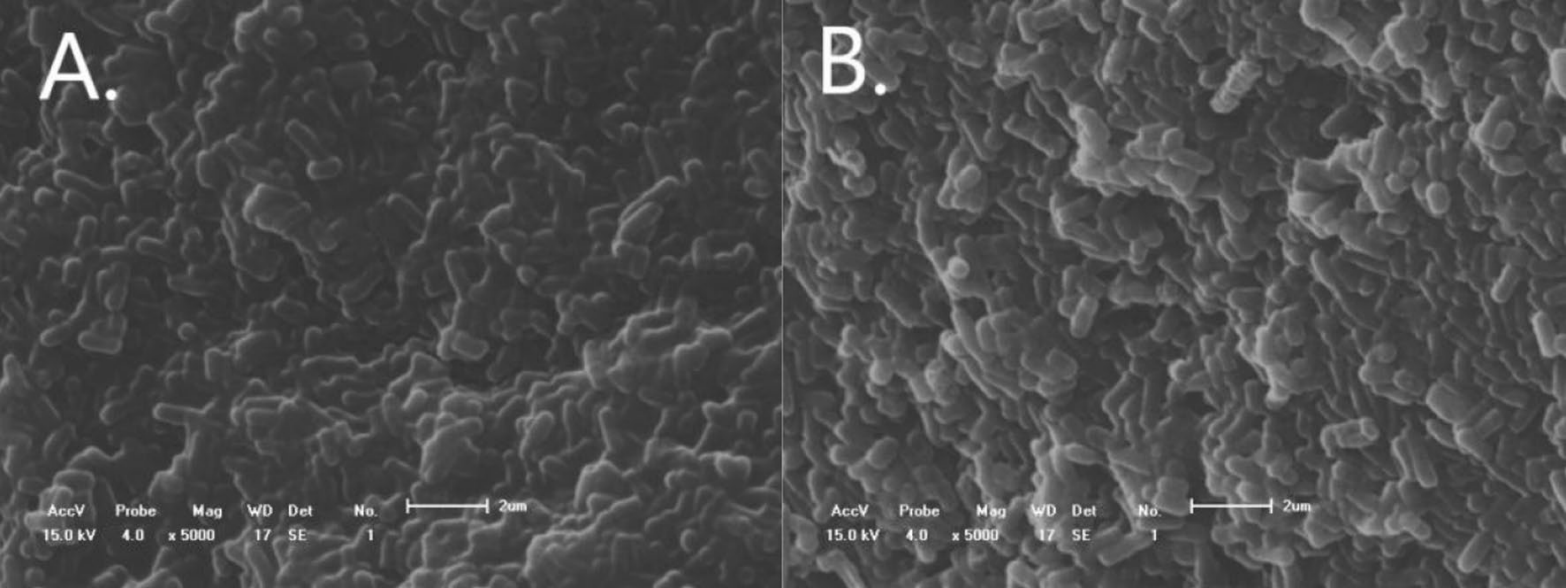
SEM images of strain XZN4 before and after Zn^2+^ adsorption. (**A**) shows the morphology of normal bacteria (5000× magnification), and (**B**) shows the morphology of bacteria after removing Zn^2+^ (5000× magnification).

#### Influence of time on the removal of Zn^2+^

The effect of time on the removal of Zn^2+^ is shown in Table 3. XZN4 strain had the fastest Zn^2+^ removal between 0 and 10 min when the removal rate reached 57.2%. At 30 min, cognitive aspiration occurred. Between 30 and 60 min, the removal process slowed down, and basically reached a state of equilibrium after 60 min. At 60 min, the removal rate of Zn^2+^ reached a maximum of 80.9%. Between 0 to 10 min, the removal process of biological passive adsorption was of functional groups on the cell wall and Zn^2+^ fast combination. After 60 min, the highest adsorption removal achieved the balance state.

**Table 3:** The ability of the bacteria to remove Zn^2+^ with time.

Time	0	10	30	60	90	120	150
Zn^2+^ removal rate (%)	0	57.2	71.5	80.9	78.6	78.52	79.1

#### Influence of temperature on the removal of Zn^2+^

The effect of temperature on the removal of Zn^2+^ is shown in Table 4. The results show that the removal rate of Zn^2+^ by XZN4 increased and then decreased with temperature. When the temperature was between 15 and 20 °C, the removal rate increased significantly. This is because the activity of the polymer in the bacterial cell wall increases with the temperature, which is conducive to the binding of heavy metal ions. When the temperature was between 20 and 35 °C, the removal rate did not change significantly, ranging from 65 to 74%. When the temperature reached 30 °C, the removal rate of Zn^2+^ reached a maximum of 84.5%. When the temperature was between 30 and 40 °C, the removal rate decreased gradually, because the activity of the polymer was seriously affected, resulting in the decrease of the stability of the complex formed by the polymer and heavy metal ions. Therefore, the optimum temperature for removal was 25–30 °C, when the removal rate was the highest.

**Table 4:** Effect of temperature on the ability of bacteria to remove Zn^2+^.

Temperature	15	20	25	30	35	40
Zn^2+^ removal rate (%)	40.5	65	76.5	84.5	74	42

### Mechanism of Zn^2+^ removal by XZN4 strain

#### SEM analysis

The scanning electron microscope (SEM) images before and after the removal of Zn^2+^ by strain XZN4 are shown in Fig. 6. The morphology of normal bacteria was short rod-shaped and full, which was consistent with the morphological characteristics of *Spinobacter*^25^. After the adsorption of Zn^2+^, the morphology of some bacteria was deformed to a certain extent, showing a shriveled shape and crystal precipitation on the surface. This may be due to the metabolites secreted by the bacteria cells in the process of Zn^2+^ adsorption, and the formation of precipitation with Zn^2+^, as well as the enhanced tolerance of the bacteria to heavy metal ions^26–28^.

#### EDS analysis

In order to test whether the cell surface observed by SEM was Zn^2+^ particles, EDS analysis was performed. Table 5 shows that XZN4 before Zn^2+^ adsorption, surface of bacteria is only carbon (C) and oxygen (O) and phosphorus (P) of the peak, strains in 100 mg/L Zn^2+^ foster growth, through the spectrum appeared the absorption peak of Zn^2+^, P^5+^ content decreased significantly, showed that bacterial cell surface combined with P^5+^ sites were replaced by a Zn^2+^, have more teichoic acid in the cell walls of bacteria, it has a strong negative charge can combine with heavy metal ions.The change of zn atomic ratio continues to increase, that is, ion exchange occurs between the two, thus improving the adsorption rate of the adsorbent. When the concentration of Zn^2+^ was 200 mg/L, it could be seen that the zinc atomic ratio did not change much, but P^5+^ continued to increase, indicating that in the case of high concentration of Zn^2+^, high concentration of Zn^2+^ might have toxic effects on bacterial cells and affect the adsorption capacity of heavy metals.

**Table 5:** Element atomic ratio before and after adsorption under different concentrations of Zn^2+^.

Element	0 mg/L Zn^2+^	100 mg/L Zn^2+^	200 mg/L Zn^2+^
C	71.92	65	76.5
O	22.45	22.47	21.11
P	2.22	2.52	3.08
Zn	0	0.98	0.15

#### FTIR analysis

**Figure 7:**
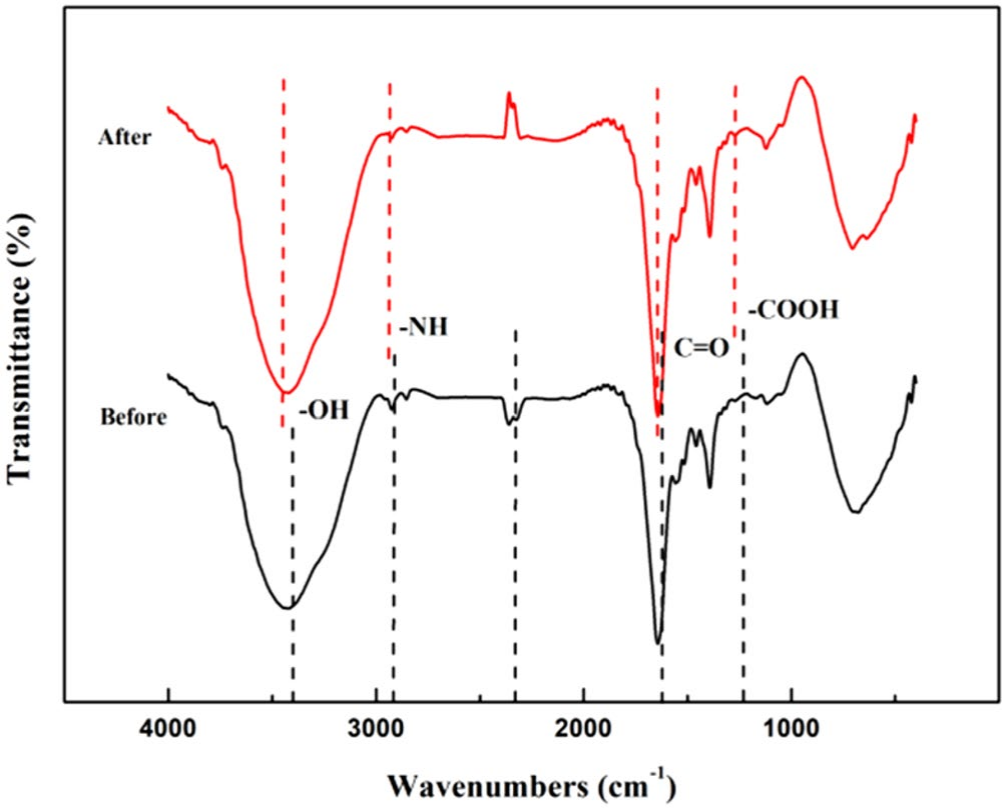
Infrared spectra of XZN4 strain before and after adsorption of Zn^2+^. The infrared spectroscopy was overlaid in the 4000–400 cm^–1^ region. The infrared spectroscopy of the bacteria was enhanced and shifted along the y-axis to increase visibility through the origin.

The infrared spectra of XZN4 strain are shown in Fig. 7. The absorption peak from 3200 cm^−1^ to 3500 cm^−1^ was wide and strong, which is the characteristic spectral peak of stretching vibration of –OH^29^. The absorption peak from 2900 to 3000 cm^−1^ was characteristic of –NH bending vibration. The absorption peak from 2000 to 2500 cm^−1^ was caused by –SH stretching vibration. Typical amide I belt (C=O stretching vibration), amide II belt (NH bending vibration and –CN stretching vibration superposition), and amide III (protein, fatty acid stretching vibration) had maximum adsorption peaks in 1625 cm^−1^, 1450 cm^−1^, and 1552 cm^−1^, respectively^30^. The absorption peak at 1250 cm^−1^ was caused by the bending vibration of the carboxyl group (–COOH) and the stretching vibration of P=O and P–O bonds in phosphate. The stretching vibration of C–N, phosphate, and sugar ring in the amine group appeared at 1073.88 cm^−1^. The absorption peak from 500 to 750 cm^−1^ was caused by the stretching vibration of O and M (metal ion) single bond region^31^. Therefore, the presence of peptidoglycan, polysaccharide, phospholipid, and other functional groups in the cell leads to the change of spectral peak, and these groups can be applied to different heavy metals.

The comparison of infrared spectra before and after the adsorption of Zn^2+^ by XZN4 strain showed that the spectral peaks of some functional groups were redshifted. The spectral peak at 3460 cm^−1^ and 2970 cm^−1^ showed slight redshift, while the stretching vibration of –OH bond and –NH bond changed, indicating that during the removal process, it might have been the result of coordination complexation between Zn^2+^ and –OH on the cell wall surface of bacteria. The disappearance of the peak at 2361.93 cm^−1^ was mainly attributed to the electrostatic attraction between Zn^2+^ and the cell wall, as well as the exchange of H^+^ or other cations. The peak shape of the spectral peak at 1625 cm^−1^ changed, indicating that C=O was involved in the removal of Zn^2+^. The absorption peak for the carboxyl group (–COOH) at 1250 cm^−1^ and the vibration peak of P=O and P–O bonds in phosphate also changed slightly, indicating that Zn^2+^ could bind to the carboxyl and phosphate on the cell surface during the removal. In addition, other absorption peaks did not change position, so these functional groups did not participate in the removal of Zn^2+^. In summary, during the Zn^2+^ removal by XZN4 strain, the hydroxyl group, –NH, C=O, carboxyl group, and phosphate on the cell surface were all involved in the removal process.

#### Adsorption isotherm analysis

**Figure 8:**
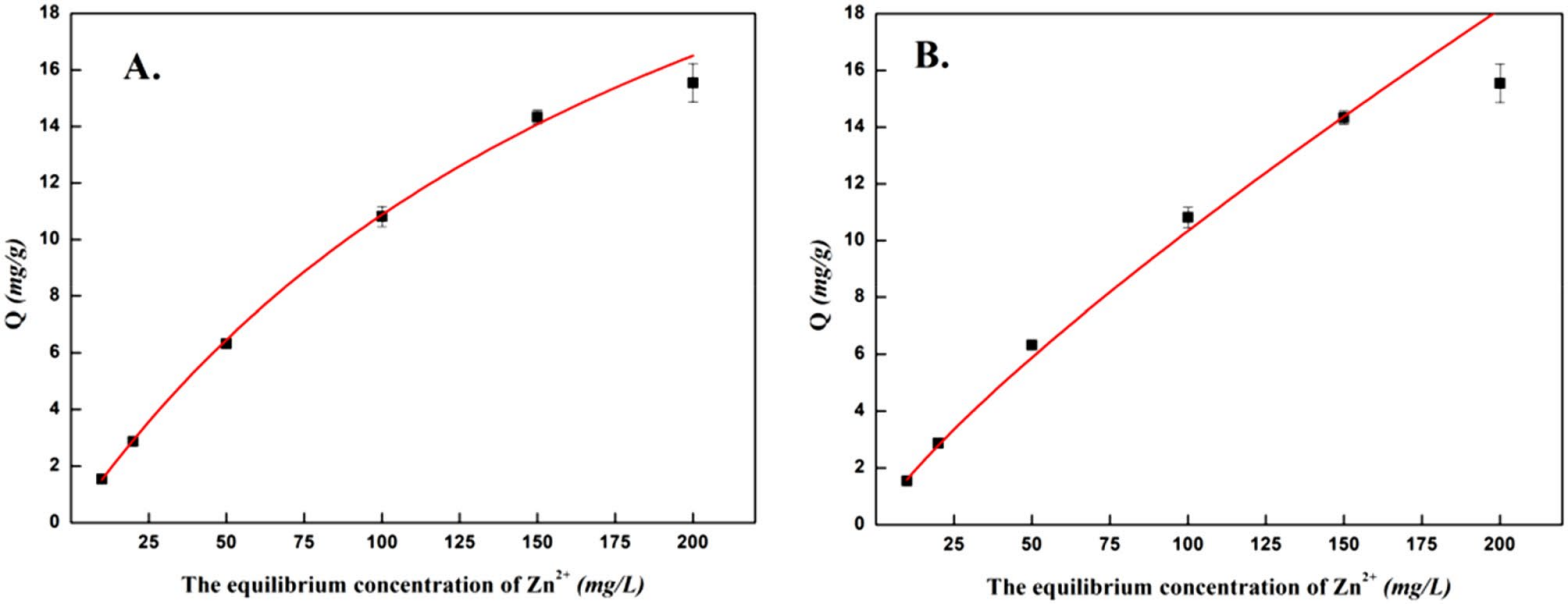
Langmuir (**A**) and Freundlich (**B**) isothermal adsorption curves of XZN4 adsorbed Zn^2+^. Q is the adsorption amount of Zn^2+^ at equilibrium.

The experimental data were fitted and analyzed using the equations of the two isothermal adsorption models. The fitting curve is shown in Fig. 8. The constants of the adsorption isotherm equation were calculated using the equation in Table 6.

**Table 6:** Isothermal equation constants and correlation coefficients.

Langmuir isothermal equation constant				Freundlich isothermal equation constant		
	q_m_	K_L_	r	K_F_	n	r
Zn^2+^	34.23429	0.00465	0.99855	0.24535	1.23108	0.99264

By comparing the isothermal equation constants of the two models, the results showed that the correlation coefficient (R) of the Zn^2+^ concentration removed from the strain was higher than that of the Langmuir equation model, and the fitting effect was better. Therefore, the removal process of XZN4 strain is more in line with the Langmuir isothermal model, indicating that the removal of Zn^2+^ corresponds to the chemical and biological removal process, and the binding sites of the removal of Zn^2+^ on the cell surface are dominated by single-molecule layer adsorption^32^.

#### Influence of inactivated bacteria on the removal effect of Zn^2+^

Live and dead cells of somatic cells were able to remove heavy metals; dead bacteria cells not affected by the heavy metal poisoning and environmental stress, the adsorption capacity is more stable. The death of the bacteria cells by the heavy metal ions adsorption process was passive, it does not depend on metabolism or energy. Therefore, the adsorption process of the strain can be distinguished by comparing the adsorption concentrations^33–36^.

The effect of sterilizing and removal of XZN4 bacteria is shown in Table 7. The results show that the removal of living cells on Zn^2+^ rate was 68.33%, and the removal rate of dead cells was 35.60%. Indicating that both living cells and dead cells have a removal effect on Zn^2+^. In particular, the removal rate of dead cells and living cells was basically the same, indicating that the adsorption process of living cells was the simultaneous action of adsorption and degradation.

**Table 7:** Comparison of the removal effects of live and inactivated bacteria on Zn^2+^ after the sample was subjected to sterilization experiments.

	Untreated	Sterilization treatment
Removal rate (%)	68.7	33.9

### Analysis of the influencing factors of XZN4 strain in livestock and poultry sewage

#### Influence of the presence of Cu^2+^ on the removal effect

In livestock and poultry sewage, Cu^2+^ and Zn^2+^ are generally concomitant. Since the amount of bacterial addition is fixed, metal ions will compete for the adsorption and binding sites of the bacteria, and the presence of Cu^2+^ will have a certain influence on the bacterial removal effect of the target Zn^2+^. It is concluded from Table 8. that when the concentration of Cu^2+^ was 10–50 mg/L, we found that the removal rate of Zn^2+^ decreased with the increase of Cu^2+^ concentration. At this time, Cu^2+^ occupied a large number of sites on the cells, and the bacteria preferred to bind to Cu^2+^. When the concentration of Cu^2+^ was 50–100 mg/L, the removal rate of Zn^2+^ increased with the increase of Cu^2+^ concentration, and the bacteria preferred to bind to Zn^2+^. When the concentration of Cu^2+^ exceeded 100 mg/L, the removal rate of Zn^2+^ decreased as the concentration of heavy metals in the solution increased and exceeded the tolerance of the bacteria. Therefore, in the range of 50–100 mg/L, the bacteria preferentially adsorbed Zn^2+^.

**Table 8:** Effect of Cu^2+^ concentration on the ability of the bacteria to remove Zn^2+^.

Cu^2+^concentration (mg/L)	0	25	50	100	150	200
Zn^2+^ removal rate (%)	80.6	65.3	43.9	33.5	68.1	27.52

#### Influence of ammonia nitrogen concentration on the removal of Zn^2+^

**Figure 9:**
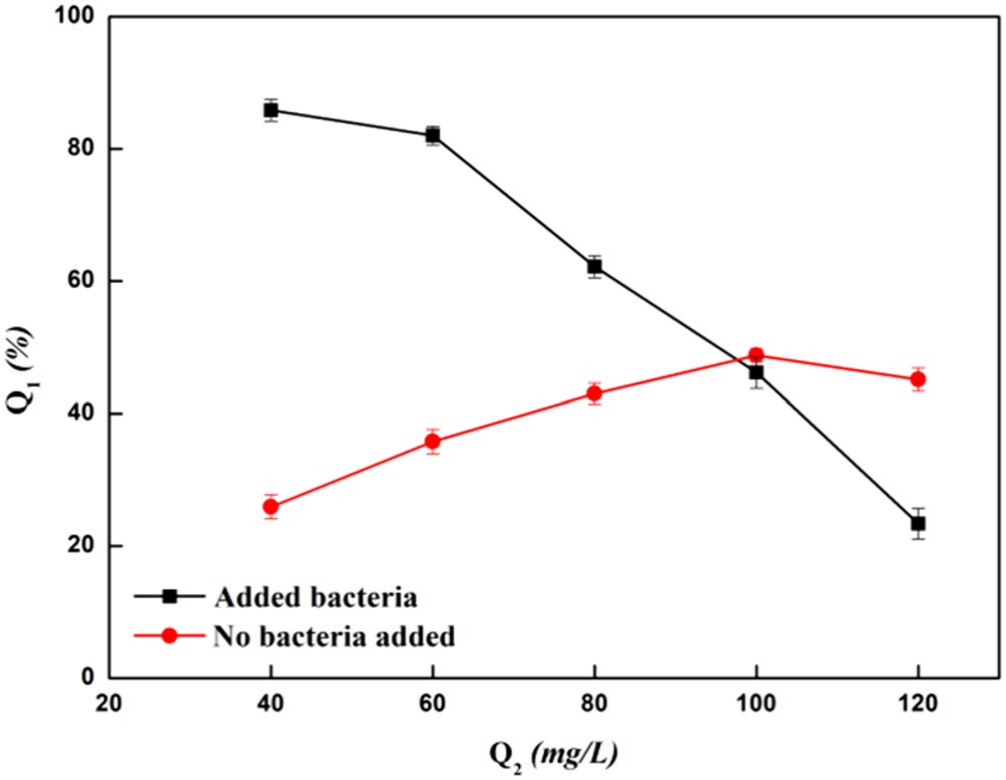
Effect of ammonia nitrogen concentration on the ability of bacteria to remove Zn^2+^. Q_1_ is the removal rate (%), Q_2_ is the concentration of ammonia nitrogen (mg/L).

The influence of ammonia concentration on the removal of Zn^2+^ is shown in Fig. 9. In the absence of bacteria, Zn^2+^ will form stable complex ions with the increase of ammonia nitrogen concentration. When the concentration reached 100 mg/L, the removal rate of Zn^2+^ was 35.33%. After the addition of bacteria, the removal rate of Zn^2+^ was significantly improved during the initial stage. With an ammonia nitrogen concentration of 60 mg/L, the removal rate was 82.03%. At this time, the adsorption of Zn^2+^ by the bacteria was not affected. However, when the ammonia nitrogen concentration reached 80–100 mg/L, the removal rate dropped from 62.17% to 46.17%, and when the ammonia nitrogen concentration was 80 mg/L, the removal rate of bacteria was even lower than that of the control group. This was because the high concentration of ammonia nitrogen inhibited the bacterial adsorption of Zn^2+^. On the other hand, a large number of complex ions formed by Zn^2+^ hindered the adsorption process. Therefore, when ammonia nitrogen concentration was less than 60 mg/L, the bacteria had the best Zn^2+^ removal effect.

#### Influence of total phosphorus concentration on the removal of Zn^2+^

**Figure 10:**
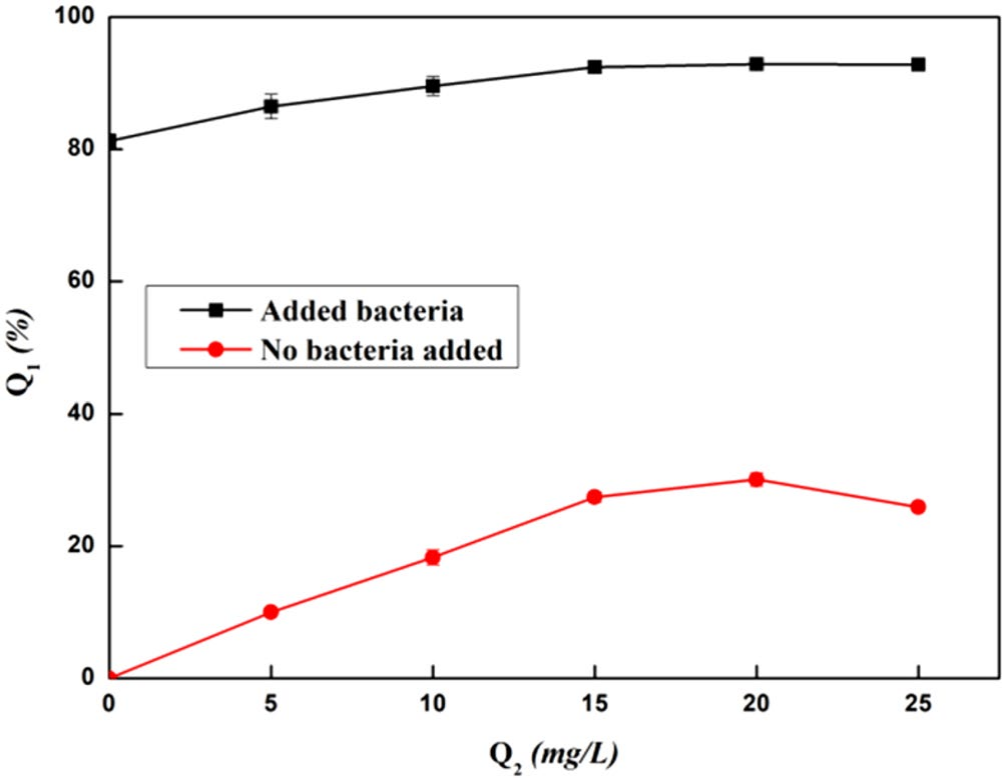
Effect of total phosphorus concentration on the ability of bacteria to remove Zn^2+^. Q_1_ is the removal rate (%), Q_2_ is the concentration of total phosphorus (mg/L).

The influence of total phosphorus concentration on the removal of Zn^2+^ is shown in Fig. 10. Phosphoric acid generally reacts with Zn^2+^ in solution. With the increase of phosphorus concentration, the removal rate also increased. After the addition of bacteria, the removal rate increased with the increase of phosphorus concentration from 81.2% at 0 mg/L to 92.9% at 20 mg/L, and stabilized after 20 mg/L. This indicates that, within a certain range, the total phosphorus concentration does not inhibit the adsorption of Zn^2+^ by bacteria, instead, it had a certain promoting effect.

#### Influence of chlortetracycline concentration on the removal of Zn^2+^

The influence of chlortetracycline concentration on the removal effect of Zn^2+^ is shown in Table 9. When chlortetracycline was not added, the removal rate of Zn^2+^ was 75.8%. After adding chlortetracycline, the removal rate of Zn^2+^ decreased rapidly due to interference with bacteria. When the concentration of chlortetracycline increased from 0.01 to 0.05 mg/L, the removal rate of Zn^2+^ decreased from 65.8% to 43.6%, and the XZN4 strain showed certain tolerance to the low concentration of chlortetracycline. With the continuous increase of chlortetracycline concentration, the removal rate of Zn^2+^ was less than 10% or in the range of 0.6–1 mg/L. When the concentration of chlortetracycline reached 2 mg/L, the removal rate of Zn^2+^ decreased to less than 1%. This indicates that the bacteria have certain sensitivity to chlortetracycline, and can normally adsorb Zn^2+^ only within 0.05 mg/L of chlortetracycline.

**Table 9:** Effect of chlortetracycline concentration on the ability of bacteria to remove Zn^2+^.

Chlortetracycline concentration (mg/L)	0.0	0.05	0.1	0.15	0.2	0.4	0.6	0.8	1.0	2.0
Zn^2+^removal rate (%)	81.2	75.6	42.3	33.5	11.1	9.82	7.68	4.62	3.58	3.02

#### Application of XZN4 for the removal of Zn^2+^ from livestock and poultry sewage

XZN4 was used to treat five groups of complex livestock and poultry sewage samples with different concentrations of Zn^2+^. The sewage was preliminarily treated, and the concentrations of chlortetracycline, ammonia nitrogen and phosphorus were respectively controlled within 0.05, 80, and 20 mg/L, respectively. The removal effect is shown in Table 10. After the removal of Zn^2+^, the concentration of Zn^2+^ was 1.30, 1.51, 1.96, 1.28, and 0.71 mg/L, with a removal rate of 85.8, 80.7, 71.3, 85.3, and 88.6%, respectively. According to the relevant guidelines of China's Farmland Irrigation Water Quality Standard (GB 5084-2005), Zn^2+^ ≤ 2.0 mg/L meets the standards for all crop types in farmland irrigation. Therefore, when the concentration of Zn^2+^ is ≤ 10.30 mg/L, XZN4 strain has direct application ability to remove Zn^2+^ to reach standard levels.

**Table 10:** Influence of XZN4 on removal of Zn^2+^ in sewage with different indicators.

Water samples	Zn^2+^ before removal (mg/L)	Zn^2+^ after removal (mg/L)	Zn^2+^ removal rate (%)
1	9.16	1.30	85.8
2	7.84	1.51	80.7
3	10.30	1.96	71.3
4	8.73	1.28	85.3
5	6.24	0.71	88.6

## Conclusions

1. A moderately Zn-resistant bacterium named XZN4 was isolated and purified from livestock and poultry feces, and confirmed to be narrow-eating *maltophilia*. Single factor analysis showed that the optimal removal conditions of Zn^2+^ by XZN4 strain were as follows: within 3 times of repeated use, pH value of 5, 100 mg/L initial concentration of Zn^2+^, 6 g/L of bacteria added, temperature of 25–30 °C, and removal equilibrium time of 60 min.
2. SEM, adsorption isothermal analysis, living and dead cells, and FTIR analysis showed that binding sites of the removal of Zn^2+^ on the cell surface were dominated by single-molecule layer adsorption. The removal process of the strain was a synergistic effect of adsorption and degradation, and the hydroxyl, –NH, C=O, and carboxyl groups, and phosphate on the cell surface were involved in binding to Zn^2+^.
3. The results showed that the ammonia nitrogen concentration was less than 80 mg/L, XZN4 strain had a good effect on the removal of Zn^2+^, and the phosphorus concentration promoted the removal of Zn^2+^ within the test range (0–25 mg/L). For XZN4 strain to maintain removal activity of Zn^2+^, the concentration of chlortetracycline has to be within 0.05 mg/L. Therefore, the strain can be applied to the treatment of livestock and poultry sewage under 80 and 20 mg/L, respectively, after preliminary treatment. Finally, XZN4 was applied to five groups of complex livestock and poultry sewage with different indicators. The results showed that the Zn^2+^ concentration after removal treatment could meet the discharge standard.

## Materials and methods

### Screening and identification of bacterial strains

Under sterilized conditions, a 0.2 mL of livestock and poultry waste samples was added to the solid medium containing Zn^2+^. Colonies were cultivated in an incubator at a constant temperature of 28 °C for 24 h, with repeated crossed separation and continuous culture enrichment until colonies were visible. A purified strain of bacteria from the solid medium was selected and named XZN4.

The total DNA of the extracted XZN4 strain was amplified by PCR, and the PCR products were sequenced and compared. The results were analyzed by Blast sequence analysis and compared with the National Center for Biotechnology Information (NCBI) nucleic acid database. The 16S rDNA sequences with high homology were selected, and a phylogenetic tree was constructed using the Neighbor Joining method using MEGA 5.0 software.

### Study of the tolerance of XZN4 strain to Zn^2+^

#### Activation and culture of XZN4 strains

Primary activation of the strain: The isolated strain was selected and inoculated into the sterilized and cooled fixed medium, which was incubated at 28 °C for 24 h.

Secondary activation of the strain: appropriate amount of the primary activated culture strains was selected by inoculation ring, injected into the sterilized and cooled liquid medium, and placed in the incubator for 24 h at 28 °C with an oscillation rate of 180 rpm/min. The expanded culture liquid was then centrifuged at 10,000 rpm/min for 50 min. The supernatant was discarded, and the bacteria at the bottom of the centrifuge tube was retained, inverted for 10 min to remove excess water and selected the wet bacteria as adsorbent.

#### Determination method

In this experiment, the concentration of Zn^2+^ was determined by flame atomic absorption spectrophotometry, and the standard curve of Zn^2+^ was drawn according to standard methods^37^. The number of microorganisms on each biochar-microbial carrier was determined using the lipid-phosphorus method^37^.

#### Determination of the Zn^2+^ tolerance of XZN4 strain

To study tolerance, 1 mL of the strain was inoculated into a sterilized liquid medium, set to 28 °C and centrifuged at 180 rpm/min under secondary activation after 24 h, with moving experiences after secondary activation of liquid bacteria. Approximately a 1% of the quantity of inoculation of Zn^2+^ concentration was set to 0 mg/L, 10 mg/L, 20 mg/L, 50 mg/L, 100 mg/L, 150 mg/L, 200 mg/L of the beef liquid medium at 28 °C, oscillating at 180 rpm/min for 48 h.

The absorbance of the culture medium was measured at 600 nm by sampling every 6 h. The growth curve of XZN4 strain at different concentrations of Zn^2+^ was plotted to observe variations with time.

### Studies on the removal conditions of heavy metal ion Zn^2+^ by XZN4 strain

#### The influence of pH value on the removal of Zn^2+^

The pH value of the adsorption solution was adjusted to 3, 4, 5, 6, 7 and 8 with 1 mol/L of NaOH and HCl. The concentration of Zn^2+^ in the solution was 100 mg/L. The bacteria medium (0.4 g) was added and placed in an oscillating incubator at 28 °C for 180 rpm/min and cultured for 150 min. The concentration of Zn^2+^ in the supernatant was determined after centrifugation.

#### The influence of the amount of bacteria input on the removal of Zn^2+^

The adsorption solution with Zn^2+^ (100 mg/L) and adjusted pH value (set to 5), received 0.2, 0.3, 0.4, 0.5, 0.6, 0.8, 1.0, and 1.2 g of the isolated bacteria, and was placed in the oscillation incubator at a constant temperature of 28 °C and 180 rpm/min for 150 min. The Zn^2+^ concentration was determined from the centrifugal supernatant.

Influence of initial concentration of heavy metal on removal conditions of Zn^2+^.

The initial concentration of Zn^2+^ was 0, 10, 20, 50, 100, 150, and 200 mg/L with adjusted pH to 5. Approximately 0.4 g of bacteria was added after centrifugation in an oscillating incubator at 28 °C for 180 rpm/min for 150 min. The concentration of Zn^2+^ in the supernatant was determined after centrifugation.

#### Influence of time on the removal of Zn^2+^

The initial 100 mg/L Zn^2+^ solution with adjusted pH value of 5 and treated with 0.4 g of bacteria was placed in an oscillating incubator at 28 °C and 180 rpm/min. The culture time was set at 0, 10, 30, 60, 90, 120 and 150 min.

#### The effect of temperature on the removal of Zn^2+^

The pH adjusted (set to 5) solution of Zn^2+^ with a concentration of 100 mg/L was treated with 0.4 g of the isolated bacteria and placed in an oscillating incubator at 180 rpm/min and cultured for 150 min. The temperature was set at 15, 20, 25, 30, 35, and 40 °C.

#### Calculation of the removal rate and removal quantity

The removal rate Q (%) and removal quantity q (mg/g) of heavy metal ions by bacteria was calculated as follows:

$$\mathrm{Q}=\frac{Ck-{C}_{i}}{Ck}\times 100\%$$

$$\mathrm{q}=(\mathrm{Ck}-{\mathrm{C}}_{\mathrm{i}})\times \frac{{\mathrm{V}}}{{\mathrm{m}}}$$

where *Ck* refers to the concentration of heavy metal ions (mg/L), *C*_*i*_ to the equilibrium concentration of heavy metal ions (mg/L), V to the volume of the solution (L), and *m* to the wet weight of the isolated bacteria (g).

### Mechanism of Zn^2+^ removal by XZN4 strain

#### SEM analysis

The bacteria before and after adsorption were treated according to the glutaraldehyde fixation method^38^, where the fixed bacteria were put into a dryer, and then sprayed with gold and observed by scanning electron microscope (SEM) (JSM-7800F, Japan).

#### Adsorption isotherm analysis

We applied the commonly used Langmuir isothermal equation and Freundlich equation as isothermal adsorption models. The equations are as follows:

Langmuir sorption isotherm equation:

$${q}_{e}=\frac{{K}_{L}{q}_{m}{C}_{t}}{1+{K}_{L}{C}_{t}}(Nonlinear\, form)$$

Freundlich function:

$${q}_{e}={K}_{F}{C}_{t}^{1/n}(Nonlinear\, form)$$

where Q_e_ represents equilibrium adsorption capacity (mg/g), Q_m_ and K_L_ are Langmuir model parameters, respectively representing maximum adsorption capacity (mg/g) and adsorption energy (mg/L)^−1^, K_F_ and n are Freundlich model parameters, respectively representing adsorption capacity (mg/g) and adsorption intensity (mg/L)^−1^.

#### Influence of inactivated bacteria on removal effect

The liquid medium was shaken for 24 h, then placed in a high temperature sterilizing pot for 30 min. The inactivated bacteria were obtained by centrifugation. Then the pH of the adsorption solution containing 100 mg/L of Zn^2+^ was adjusted to 5. Approximately 0.4 g of the inactivated bacteria was added and placed in an oscillating incubator at 28 °C, 180 rpm/min, and cultured for 150 min. After centrifugation, the concentration of Zn^2+^ in the supernatant was determined.

#### Infrared spectrum analysis

A stock solution of 20 mL was prepared with an initial concentration of heavy metals of 100 mg/L, bacteria concentration of 8.0 g/L, and adjusted pH to 5, was put in oscillation incubator at 28 °C and 180 rpm/min, and cultivated for 24 h. The centrifuged bacteria was freeze-dried for 48 h, grinded in the agate mortar with KBr powder for 5 min, and blended. Detection was performed under the Fourier transform infrared spectrometer (Nicolet iS10, United States of America).

### Influence factors on the removal of Zn^2+^ from livestock and poultry sewage by the XZN4 strain

#### The influence of Cu^2+^ on the removal efficiency

The standard solution with 100 mg/L of Zn^2+^, pH adjusted to 5, and added 0.4 g of isolated bacteria, was treated with Cu^2+^ at concentrations of 10, 20, 50, 100, 150, and 200 mg/L, placed in the oscillation at a constant temperature of 28 °C, 180 rpm/min, and cultivated for 150 min. The Zn^2+^ concentration was determined after centrifugation.

#### The influence of ammonia nitrogen concentration on the removal of Zn^2+^

The standard Zn^2+^ solution (100 mg/L) with 0.4 g of isolated bacteria and adjusted pH (set to 5), was added to the reserve liquid ammonia nitrogen, with an ammonia nitrogen concentration of 40, 60, 80, 100, 120 mg/L, placed in the oscillation incubator at a constant temperature of 28 °C, 180 rpm/min, and cultivated for 150 min. The Zn^2+^ concentration was measured in the supernatant after centrifugation.

#### The influence of total phosphorus concentration on the removal of Zn^2+^

A 0, 5, 10, 15, 20, 25 mg/L concentration of phosphorus reserve was added to a 100 mg/L solution of Zn^2+^ with a pH adjusted to 5 and 0.4 g of isolated bacteria, placed in the oscillation incubator at 28 °C, 180 rpm/min, and cultivated for 150 min, after which the Zn^2+^ concentration was determined.

#### The influence of chlortetracycline concentration on the removal effect

The solution with Zn^2+^ at a concentration of 100 mg/L with adjusted pH set to 5, and 0.4 g isolated bacteria, was treated with chlortetracycline stock solution, at concentrations set to 0, 0.01, 0.05, 0.1, 0.2, 0.4, 0.6, 0.8, 1, 2 mg/L, placed in the oscillation incubator at 28 °C, 180 rpm/min, and cultivated for 150 min. Zn^2+^ concentration was determined in the supernatant after centrifugation.

#### Application of *Stenotrophomonas maltophilia* in the treatment of livestock and poultry sewage

Five samples of livestock and poultry sewage treated by initial precipitation were collected (taken from a livestock and poultry sewage plant in Changchun). The specific indicators are shown in Table 11.

**Table 11:** Specific indicators of tested water samples.

	pH	Zn^2+^ (mg/L)	Ammonia nitrogen (mg/L)	Total phosphorus mg/L)	Chlortetracycline (mg/L)
1	5.89	9.16	62.3	7.4	0.02
2	6.72	7.84	74.5	6.7	0.03
3	7.11	10.30	86.3	8.3	0.04
4	6.44	8.73	66.8	7.7	0.02
5	6.13	6.24	72.4	5.4	0.01

0.4 g centrifuge bacteria were added to 100 mL water sample, and placed in an oscillating incubator at 28 °C, 180 rpm/min, and cultured for 150 min. After centrifugation, the concentration of Zn^2+^ was determined.
